# Distribution and Morphology of Calcium-Binding Proteins Immunoreactive Neurons following Chronic Tungsten Multielectrode Implants

**DOI:** 10.1371/journal.pone.0130354

**Published:** 2015-06-22

**Authors:** Marco Aurelio M. Freire, Jean Faber, Nelson Alessandretti M. Lemos, Jose Ronaldo Santos, Pedro França Cavalcanti, Ramon Hypolito Lima, Edgard Morya

**Affiliations:** 1 Edmond and Lily Safra International Institute of Neurosciences (ELS-IIN), Santos Dumont Institute, Macaiba, RN, Brazil; 2 Laboratory of Neuroengineering, Department of Science and Technology, Federal University of São Paulo (UNIFESP), Sao Jose dos Campos, SP, Brazil; 3 Laboratory of Neuroscience, Department of Biosciences, Federal University of Sergipe (UFS), Itabaiana, SE, Brazil; 4 Memory Studies Laboratory, Department of Physiology, Federal University of Rio Grande do Norte (UFRN), Natal, RN, Brazil; 5 Associação Alberto Santos Dumont para Apoio a Pesquisa, Sirio-Libanes Hospital, São Paulo, SP, Brazil; Tokyo Medical and Dental University, JAPAN

## Abstract

The development of therapeutic approaches to improve the life quality of people suffering from different types of body paralysis is a current major medical challenge. Brain-machine interface (BMI) can potentially help reestablishing lost sensory and motor functions, allowing patients to use their own brain activity to restore sensorimotor control of paralyzed body parts. Chronic implants of multielectrodes, employed to record neural activity directly from the brain parenchyma, constitute the fundamental component of a BMI. However, before this technique may be effectively available to human clinical trials, it is essential to characterize its long-term impact on the nervous tissue in animal models. In the present study we evaluated how chronic implanted tungsten microelectrode arrays impact the distribution and morphology of interneurons reactive to calcium-binding proteins calbindin (CB), calretinin (CR) and parvalbumin (PV) across the rat’s motor cortex. Our results revealed that chronic microelectrode arrays were well tolerated by the nervous tissue, with recordings remaining viable for up to 6 months after implantation. Furthermore, neither the morphology nor the distribution of inhibitory neurons were broadly impacted. Moreover, restricted microglial activation was observed on the implanted sites. On the whole, our results confirm and expand the notion that tungsten multielectrodes can be deemed as a feasible candidate to future human BMI studies.

## Introduction

Neuropathological conditions, such as brain and spinal cord injuries, are currently the leading causes of disabilities worldwide, affecting severely the life of its sufferers [1–5] and imposing a significant socioeconomic burden to the health care system [6,7]. One of the main medical challenges in this field deals with the development of therapies capable of ensuring a better quality of life for those suffering from devastating levels of body paralysis. Any solution provided, however, has to face the major challenge of reestablishing both sensory and motor functions lost after a brain lesion. Brain-machine interfaces (BMI), which provide a new way for establishing direct communication between the brain and an external device, such as computers and robotic limbs [8,9], offers such a possibility.

Chronic multielectrodes implants define the fundamental component of an invasive BMI. Their use ensures an accurate assessment of the neuronal activity in awake, behaving animals [10–12], representing a key tool for enhancing our general understanding of the neurophysiological principles that underlie the interactions of large populations of neurons. Nevertheless, before microwire arrays can be effectively available to human clinical trials, it is necessary to characterize their impact on the nervous tissue in animal models, particularly in the cortex. In this prospect, investigation becomes even more relevant when considering that one of the most essential prerequisite for any invasive BMI is its capability to remains functional through many years after the surgery for implantation of recording probes. Such longevity should maximize both the recording of stable high quality action potentials from hundreds to thousands of neurons and the preservation of the tissue surrounding the implant. This latter goal is critical if one intends to avoid structural and physiological alterations of brain tissue that could compromise the device’s performance and further complicate the patient’s neurological state.

The basic functions of the brain result from dynamic interactions among distinct groups of neurons [13–15], ultimately emerging as all sort of refined cognitive functions and complex behaviors we are able to perform, including the aptitude to control a robotic prosthesis [16]. According to their physiological properties, there are two major groups of neurons: (I) excitatory cells, the main class of neurons (mostly represented by the pyramidal cells), that use glutamate as their chief neurotransmitter and approximately corresponds to 75–80% of the entirety of neurons [17], and (II) inhibitory interneurons (also known as local circuit neurons), corresponding to the remaining 20–25%, which can be defined as cells whose somas, axons and dendrites are entirely located inside the cerebral cortex [18]. The main feature of this latter cell group is the establishment of connections only with nearby neurons. As such, interneurons act as modulators of local cortical circuits [19]. γ-aminobutyric acid (GABA) is the chief neurotransmitter of inhibitory interneurons, which, in turn, are represented by a quite diverse population of cells, with distinct morphological, neurochemical and physiological features [20]. Immunohistochemistry for the calcium-binding proteins (CBP) calbindin (CB), calretinin (CR), and parvalbumin (PV) reveals the existence of morphologically heterogeneous classes of GABAergic interneurons, which are involved in a variety of complex cortical circuits, depending on cortical area or layer where they are located [21].

In a previous study we characterized some aspects of the impact caused by tungsten microelectrode arrays in the motor cortex of chronically implanted rats [22]. Here, we set out to extend our tissue analysis, evaluating the general distribution and morphology of the CBP-reactive neurons in the rat’s motor cortex following chronic multielectrode array implant. As an accessory data, we also aimed to characterize the level of inflammatory response induced by chronic presence of electrodes through a selective microglial marker (Iba1), since these cells are directly involved in the regulation and maintenance of the nervous system homeostasis [23]. Furthermore, we evaluated some aspects concerning the temporal variation of electrophysiological signals in function of signal-to-noise ratio (SNR) obtained during chronic cortical recordings, comparing data from first and last week of recording in all time points analyzed (1, 3 and 6 months of implantation).

## Material and Methods

### Animals and experimental design

Fifteen adult brains of male Wistar rats (320±20g) were used. All experimental procedures were in strict accordance with the NIH Guide for the Care and Use of Laboratory Animals (NIH Publications No. 80–23), approved by Institutional Review Board (IDs # 04/2009 and 13/2011), and under the knowledge and agreement of Scientific Direction of ELS-IIN. All efforts were made to avoid any animal suffering and to reduce the number of subjects used.

**Fig 1:**
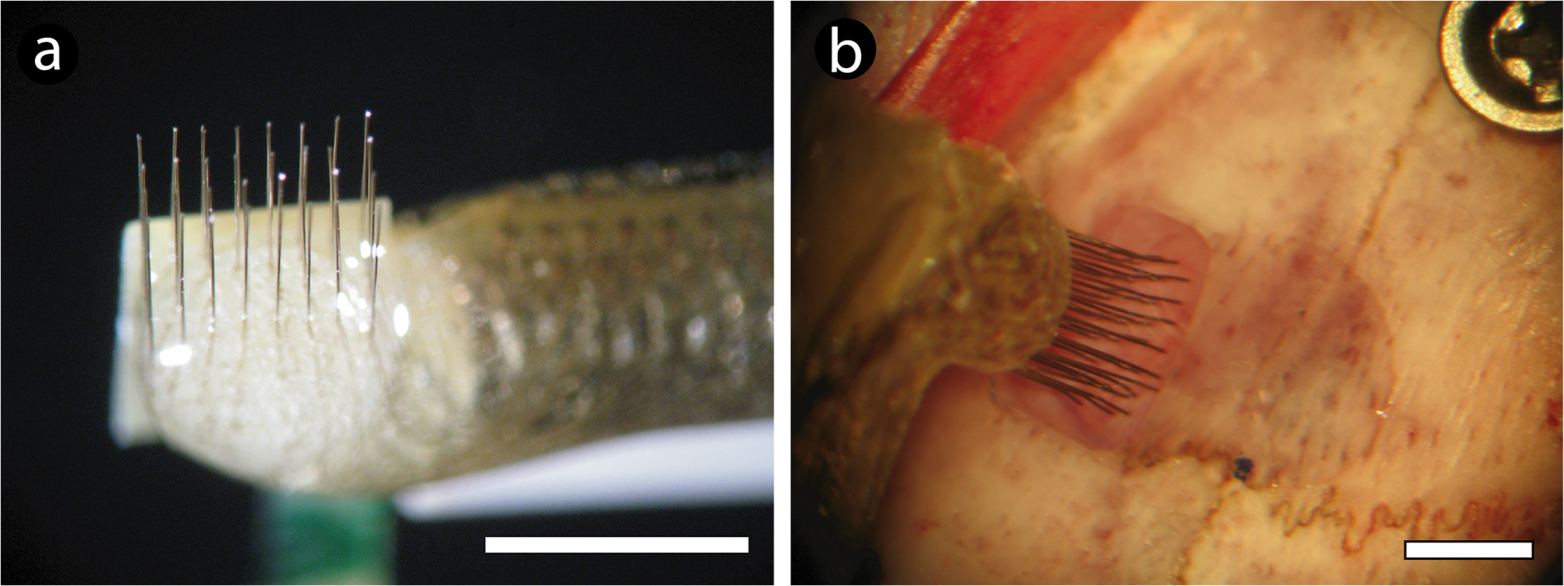
Multielectrode apparatus, coordinates adopted to implant and surgical procedure. (a). General aspect of a 32 channel microelectrode array. (b). Details of the electrode array being implanted through the skull window on primary motor cortex, relative to bregma (marked with a black dot). Scale bars: 3.5 mm (a and b).

The present study follows the same proposal adopted in our previous report concerning a suitable understanding on the impact of multielectrodes arrays in the rat’s brain [22]. Accordingly, the experimental design was quite similar. In brief, surgeries for multielectrode implantation were performed in rats deeply anesthetized with 100 mg/kg of ketamine chlorhydrate and 5 mg/kg xylazine chlorhydrate (i.p.). The animals were placed in a stereotaxic head holder, and a small craniotomy was made over the implant target area (primary motor cortex) (Fig 1). Each animal was slowly implanted with multielectrode arrays, manufactured as follows: Insulated tungsten wires (50-μm microwire diameter, 1.5 MOhm at 1.0 KHz, California Fine Wire Co., Grover Beach, CA, USA; Catalog number #100211, IS coating) were cut into 32, 5-cm segments. Each wire was soldered into separate slots in an Omnetics connector (Omnetics Connector Corp., Minneapolis, MN, USA) using a digital soldering station with a micro soldering pencil (Weller WD1, 0.25mm tip) in order to assemble an array of 4x8, with 500 μm spacing (Fig 1). The tip of the electrode was cut with a stainless steel precision surgical scissor, being visualized in a surgical magnifier (Zeiss Stemi 2000 Stereo Microscope, Carl Zeiss, Göttingen, Germany). The array was subsequently implanted in the motor cortex using the following coordinates (in millimeters relative to bregma): 0.5–4.0, anteroposterior (AP); 1.5–3.0, mediolateral (ML); 1.8–2.0, dorsoventral (DV) [24]. Stainless steel screws and dental acrylic were used to hold the implant. The ground stainless steel wire was soldered to a screw. After 7 days to surgical recovery, animals started to be recorded weekly. Three survival time groups were then defined, according to the total time of recording: 1, 3, and 6 months after initial implantation.

### Electrophysiological recordings

A 32-channel multi-neuron acquisition processor (MAP, Plexon Inc., Dallas, TX, USA) was used for spike recording and sorting in a weekly basis, for 30–45 min in rats freely behaving in their cages. Online spike sorting was conducted with the help of the SortClient 2002 software (Plexon Inc., USA). A maximum of 4 neuronal action potentials per channel were sorted online and validated by offline analysis (Offline Sorter 2.8, Plexon Inc., USA) according to some criteria, as previously described [22]: voltage thresholds, amplitude distributions, signal-to-noise ratio, waveform shapes, and principal component analysis. The temporal variation of the electrophysiological pattern in each group during its time of implant (increase or decrease of neural signal) was evaluated by comparing neural signal collected in first and last week of recording.

### Perfusion and tissue processing

After the last recording section for each group, animals were deeply anesthetized with 5% isofluorane, overdosed with sodium thiopental (90 mg/kg) and then perfused intracardially with 0.9% warm heparinized saline (Roche Pharmaceuticals, NJ, USA) (2ml/1000ml) followed by 4% cold paraformaldehyde in 0.1M phosphate buffer (PB), pH 7.4 (Sigma Company, St Louis, MO, USA). Brains were then removed from the skull and immersed in 20% sucrose in 0.1M PB for 12h. The tissue was then frozen in a embedding medium (Tissue Tek, Sakura Finetek, Japan) and sectioned in the coronal plane at 30μm in a cryostat (Carl Zeiss Microm HM 550, Jena, Germany). The resulting sections were mounted on electrically charged glasses (Super Frost Plus–VWR International, Radnor, PA, USA) and submitted to immunohistochemistry to CBP as follows: sections were pre-incubated in a 1% hydrogen peroxide solution in methanol during 20 minutes to remove endogenous peroxidase activity, being then rinsed in 0.05% phosphate buffer-Tween (PB-T) for 5 minutes and incubated in 10% horse normal serum (diluted in 0.1M PB; Vector Laboratories, Burlingame, CA, USA) for 30 minutes in order to block non-specific binding. After, sections were incubated during 16h in CB, CR and PV primary antibodies (1:1000, diluted in 1% horse normal serum; Swant, Bellinzona, Switzerland) at 18°C, washed in PB-T by 6 minutes (2x, 3min each), incubated with a biotinylated horse anti-mouse secondary antibody (1:200, diluted in 0.1M PB; Vector Laboratories, Burlingame, CA, USA) for 2 h, washed in PB-T during 10 minutes (2x, 5min each), and then incubated in avidin-biotin-peroxidase solution (Vectastain Standard ABC kit, Vector Laboratories, USA) for 1h. Slides were then placed in a solution containing 0.03% 3,3’ diaminobenzidine tetrahydrochloride (DAB) (Sigma Company, USA) and 0.001% hydrogen peroxide in 0.1M PB, dehydrated through a series of graded alcohols and coverslipped with Entellan (Merck, Darmstadt, Germany). Further, some sections were immunostained using fluorescent labels, in which horse normal serum was replaced by 10% goat normal serum, and the secondary biotinylated antibody was replaced by Alexa Fluor 488-conjugated goat anti-mouse secondary antibody (1:700, diluted in 1% goat normal serum; Invitrogen, Grand Island, NY, USA) for 2h. In order to certify the specificity of the labeling the primary antibodies were replaced by normal serum in some randomly selected sections. CB, CR and PV are monoclonal antibodies that react specifically with parts of its target protein (the first 4 EF-hands domains in calretinin, the calbindin D-28k and the ^45^Ca-binding spot of parvalbumin) that are present only in neurons, not cross-reactive with glial cells.

To reveal the general pattern of microglial activation around each microelectrode’s implant site, additional sections were stained to Iba1 using fluorescent labels (Iba 1 is a protein specifically expressed in microglial cells, not cross-reactive with neurons and astrocytes). In brief, sections were washed during 10 minutes in PB-T and pre-incubated in 10% goat normal serum during 30 minutes. Thereafter, the sections were incubated during 24h with Iba1 primary antibody (1:500, diluted in 1% goat normal serum; Wako, Osaka, Japan) at 18°C. Sections were then washed in 0.1M PB during 5 minutes and incubated with Alexa Fluor 555-conjugated goat anti-rabbit secondary antibody (1:700, diluted in 1% goat normal serum; Invitrogen, Grand Island, NY, USA) for 2h. Finally, the sections were mounted using Vectashield mounting medium for fluorescence (Antifade solution) (Vector Laboratories, USA).

Some sections were dehydrated and mounted as previously described and stained with cresyl violet (Nissl staining) to identify the exact location of the electrode tracks.

### Qualitative and quantitative analysis

For qualitative analysis, digital images were obtained with a CX9000 camera (MBF Biosciences, USA) attached to an optical microscope (Nikon Eclipse 80i, Tokyo, Japan- 10x and 20x objectives). Fluorescent images were obtained in a Carl Zeiss Laser Scanning Microscope (LSM 710, 10x and 40x objectives, Carl Zeiss, Jena, Germany). The contrast, and/or brightness of pictures were adjusted using Photoshop CS6 software (Adobe Systems Inc., San José, CA, USA).

To evaluate quantitatively the distribution of CBP-reactive neurons, we counted the total number of cells labeled by all markers (CB, CR and PV) in the region around electrode tracks in the three survival time groups (1, 3 and 6 months of implant) using the Neurolucida system (MBF Biosciences Inc., Williston, VT, USA). For each marker, we quantified 3 sections per animal, sampling tissue from regions where the electrode tracks could be unequivocally observed, covering 500μm of tissue in the anteroposterior axis. The cell density (cells/mm^2^) was estimated using an automatic grid from the Neurolucida program. The hemisphere contralateral to the implant was adopted as an intrinsic control in all animals. For the statistical comparison among groups we used both a non-parametric Kruskal-Wallis test and Spearman correlation followed by the Bonferroni *post hoc* test with significance level set at 95% (α = 0.05). Average values were referred to as mean ± standard error of the mean (SEM). When expressed in boxplots, the values were presented as median values with indication of the 25th to 75th percentiles.

### Electrophysiological signal analysis

The yield of electrophysiological signals recorded from chronically implanted microwire arrays was assessed by comparing the total number of neuronal units observed in the first and last recording sessions of a given period (1, 3, or 6 months). In addition, in order to measure the quality of these recordings we evaluated the signal-to-noise ratio (SNR), a measure employed to compares the level of a well-defined neural signal to the level of background noise. The SNR was calculated using the equation 20*log_10_ (RMS[corr_signal/corr_surrogate]), where *corr_signal* corresponds to the auto-correlation of the temporal signal of each neuron given by the firing rate matrix (binned with 1 second) and *corr_surrogate* corresponds to the cross-correlation of temporal signal and its surrogate, which was generated by using a random shuffling of the firing rate matrices columns based on an uniform distribution (RMS: Root Mean Square) [25,26]. After the SNR measurement for each neuron, the average value in every group (1, 3 and 6 months of signal recording) was then calculated. For each animal there was an average and a standard deviation of the SNR. The standard deviation was used as a threshold to digitalize the SNR—for every point above the *std* was considered as “1” and every point below *std* was considered as “0”. Adopting this procedure we were able to eliminate possible spurious variations and emphasize on the main differences among the groups.

Furthermore, in order to evaluate the degree of signal quality, we defined six different thresholds based in the strength of the signal obtained on Plexon MAP box: 0 (no signal); 0.2; 0.4; 0.6; 0.8; and 1 (maximum SNR ratio). Neurons presenting a signal with a SNR index, as measured through the abovementioned formula, higher than 0.5 were considered of good quality. This procedure was applied in every group of animals (1, 3 and 6 months of recording), allowing us to evaluate the signal quality of the recorded units over time. Mean and variance were calculated using the standard deviation as threshold (SNR>std(SNR) = 1; and SNR<std(SNR) = 0) in Matlab 2008 software (The MathWorks Inc., Natick, MA, USA).

## Results

### Quality of the electrophysiological signal over time

**Fig 2:**
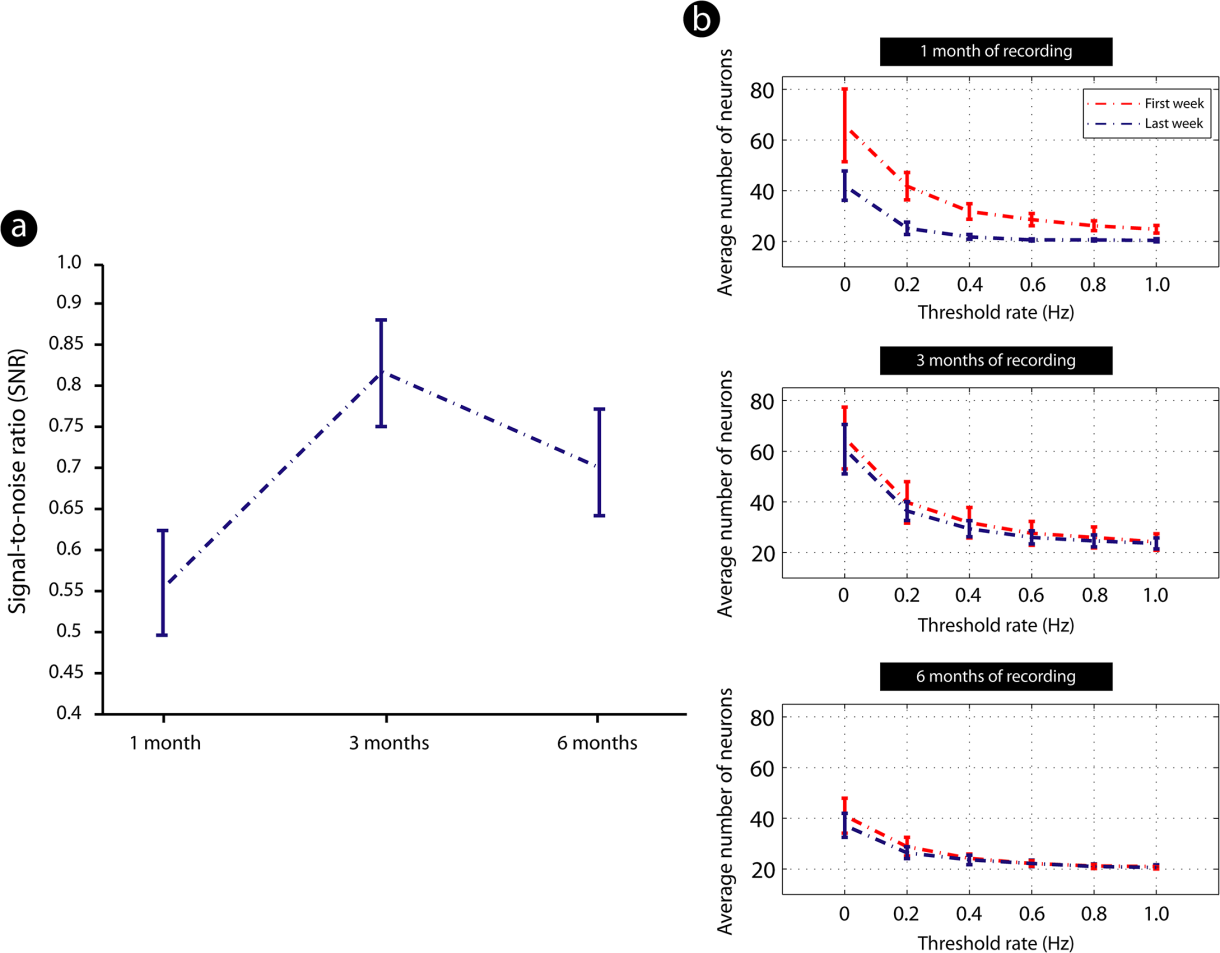
Signal-to-noise ratio (SNR) of cortical neurons recorded from all animals across distinct survival times. (a). Measurement of the SNR average among all time points revealed an increase in this parameter after 3 months of implant as compared to 1 month of implantation. In latter survival time (6 months after the array implant), quality of recordings decreased as compared to previous time point, though a good SNR still had been identified (standard deviation threshold: SNR>std(SNR) = 1; and SNR<std(SNR) = 0) (*n* = 5 animals by time point); values expressed as mean ± SEM. (b). Temporal variation of the neuronal number in each group during its time of implant, comparing the first and the last week of array implantation in all survival times, in relationship to Threshold rate (Hz) adopted to assess SNR (see Material and Methods). There was an improvement in the number of neuronal units from 1 month to 3 months of implant. In latter survival time (6 months of implant) number of recorded cells decreased (*n* = 5 animals by time point).

There was an increase in SNR after 3 months of implant as compared to 1 month of implantation (Fig 2). In latter survival time (6 months after the array implant), there was a trend towards a decrease in the recording quality as compared to 3 months survival time, although a good SNR could still be obtained (Fig 2A). We also evaluated the temporal variation of the neuronal number in each group during its time of implantation, comparing the first and the last week of implant in all survival times. There was an improvement of the number of neuronal units recorded 3 months after implant as compared to 1 month of implant. In latter survival time (6 months of implant) the number of recorded cells decreased (Fig 2B).

### General aspect of implant sites

**Fig 3:**
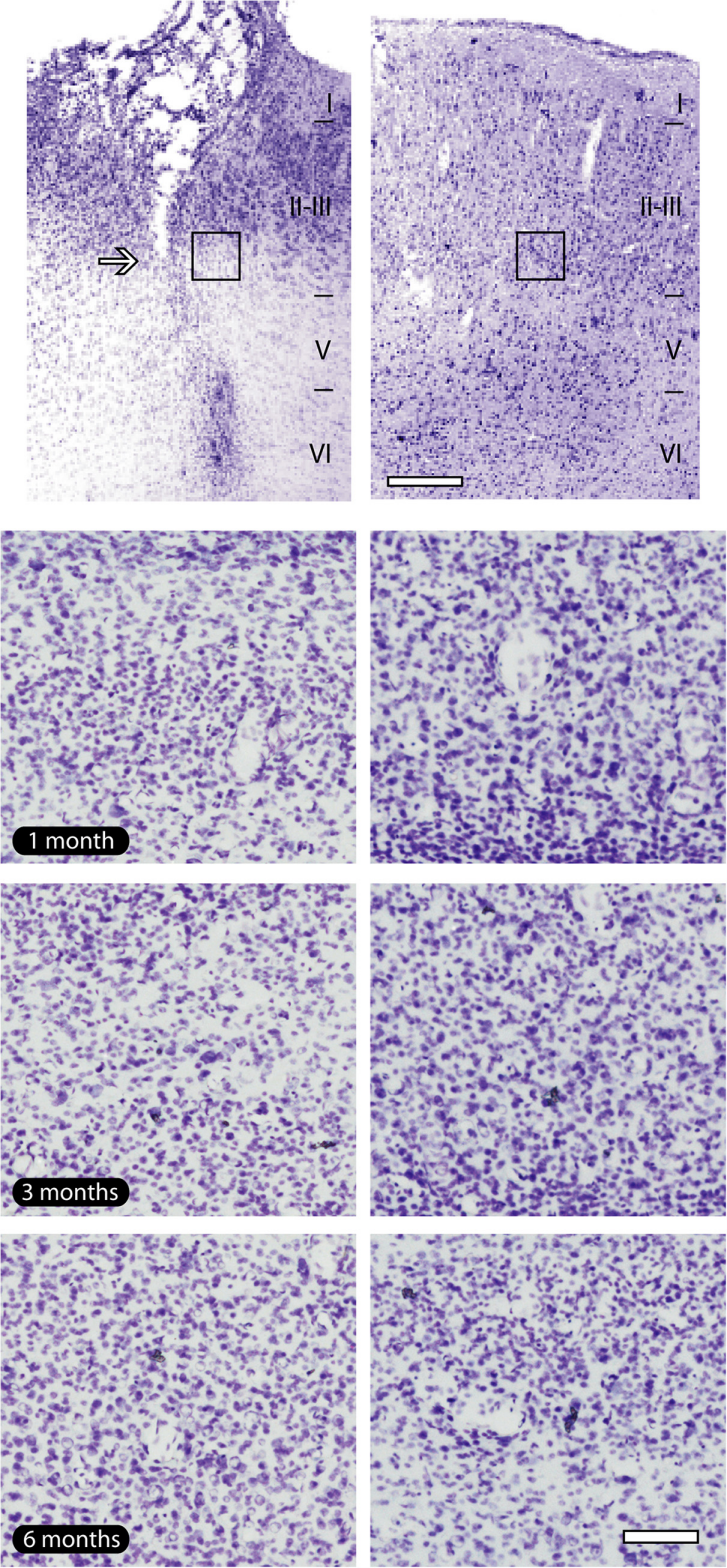
General pattern of implant sites. (a). Nissl staining shows the well-defined location of the electrode track (arrow), revealing a small loss of tissue. (b). It was possible to attest the absence of vacuolization and pyctonic profiles in all time points evaluated. The tissue around electrode implants presented a similar aspect to their contralateral counterpart. Black squares in lower magnification pictures indicate the regions where enlargements were obtained in all groups. Left column of the figure: implanted hemisphere; right column of the figure: contralateral hemisphere, adopted as control. Scale bar: 100 μm (lower magnification); 200 μm (enlargements).

The precise location of the microwire array implants was identified in all animals at all survival times (Fig 3). Nissl staining revealed a minimal tissue loss in the implant site, restricted to the exact location of electrodes, with preservation of cell bodies near the microelectrode track and the absence of vacuolization, an event usually observed after milieu alterations (Fig 3).

### Neuronal distribution across implanted sites

**Fig 4:**
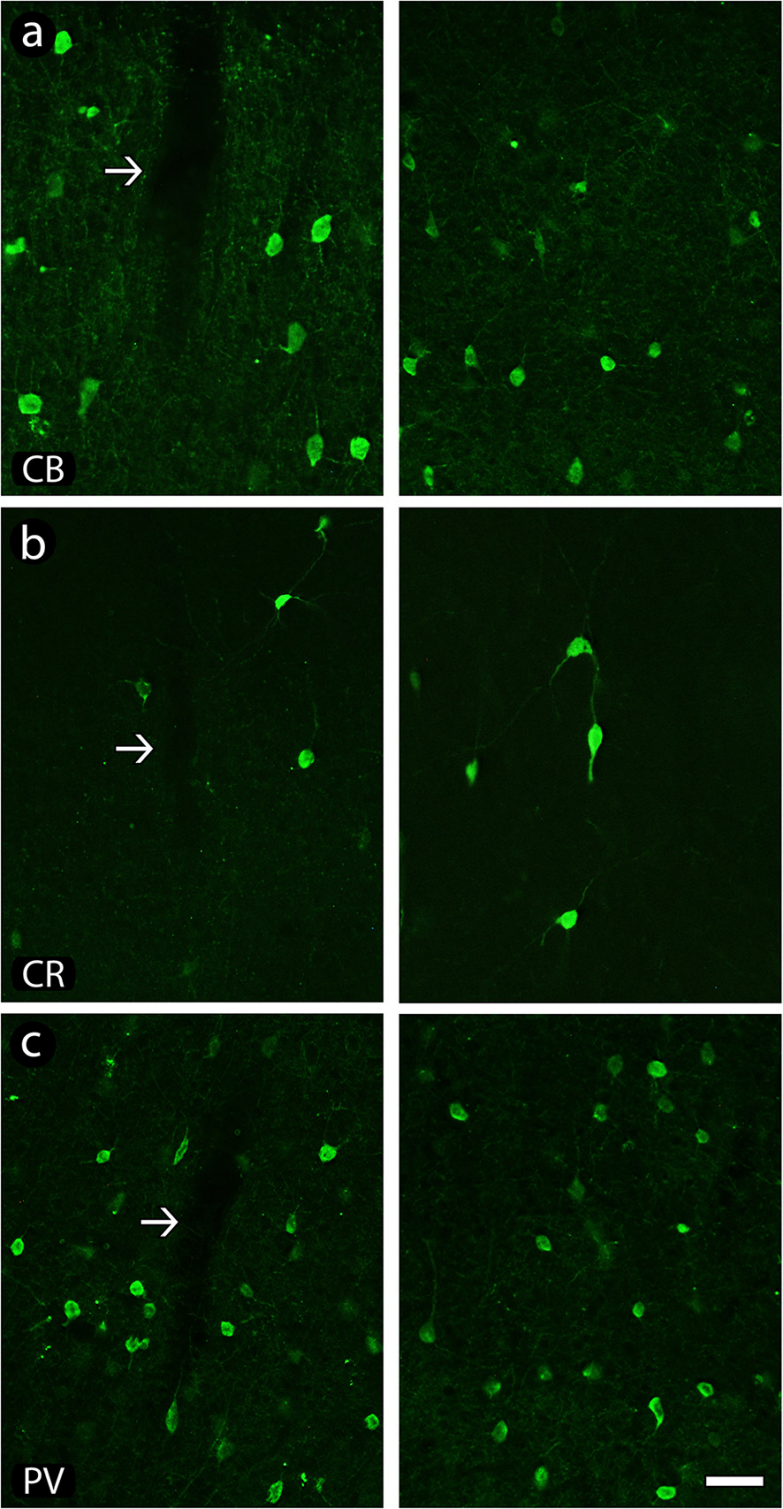
Calcium-binding proteins immunoreactivity across implanted sites. Chronic presence of electrodes did not affect the general cell distribution of calbindin (CB)- (a), calretinin (CR)- (b) and parvalbumin (PV)- (c) reactive neurons even after 6 months of implant. Left column of the figure: implanted hemisphere (arrows point to electrode tracks); right column of the figure: contralateral hemisphere, adopted as control. Scale bar: 100 μm.

In general, the distribution of both CBP-reactive neurons in the implanted tissue was similar to those seen in its contralateral counterpart, except in the exact location of electrode track, where a small loss of tissue can be noticed (arrows in Fig 4).

Calbindin-reactive neurons (CB) were found distributed across all layers of the motor cortex except in layer I, but more noticeably across layers II and III, with the pattern of cell body labeling varying from weakly to intensely reactive. The neuropil across supragranular layers was more intensely reactive than in other layers, revealing the labeling of extended, vertically oriented bundles of immunoreactive processes. Both their general morphology and distribution were not affected by electrode implant (Fig 4A).

Calretinin-reactive neurons (CR) were mainly concentrated across supragranular layers (except layer I), although some cells also could be found scattered across infragranular layers. In opposition to seen in CB, the immunoreactivity of CR-labeled neurons was more homogeneous. Similar to CB, distribution of CR-reactive cells was not altered across implanted areas (Fig 4B).

Parvalbumin-reactive (PV) neurons were located in all layers of the motor cortex, except in layer I. Similar to CR, there was a homogeneous pattern of neuropil labeling across all cortical layers in both implanted and contralateral hemispheres. A group of less-reactive cells could be discerned. Similar to abovementioned to CB and CR, the presence of electrode even after 6 months in the tissue did not alter the distribution of PV-reactive cells (Fig 4C).

**Fig 5:**
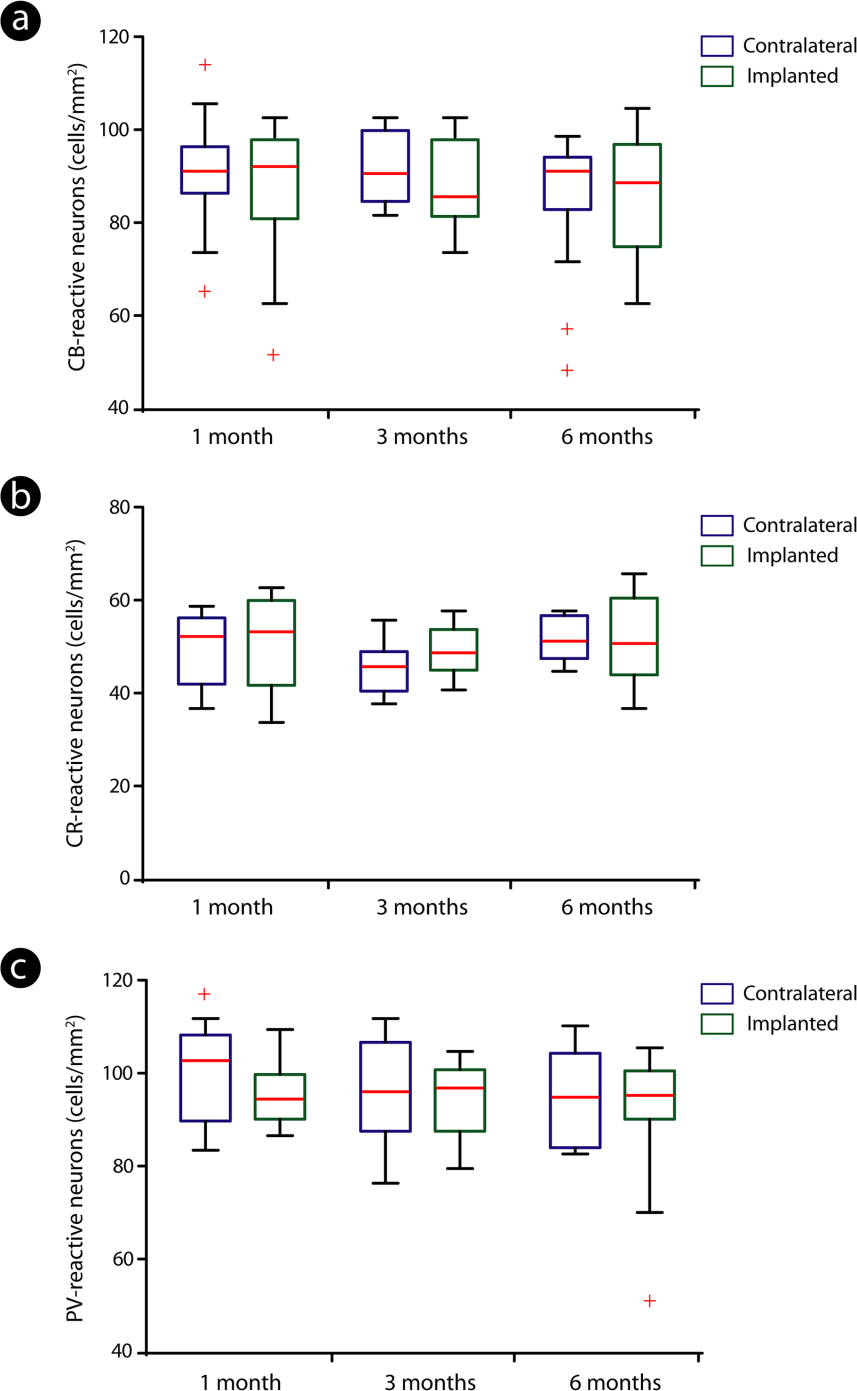
Distribution of calcium-binding proteins-reactive neurons across implanted and contralateral hemispheres. Quantitative analysis revealed that the number of calbindin (CB)- (a), calretinin (CR)- (b) and parvalbumin (PV)-reactive neurons (c) did not differ significantly when implanted and contralateral sides were compared. Distinct colors (blue: contralateral hemisphere; green: implanted hemisphere) were adopted to provide a better identification of both control and implanted groups in all time points evaluated. The boxes correspond to the 25th and 75th percentiles (bottom and top of the boxes, respectively), the median values are indicated by the red horizontal line inside each box, the whiskers point out the minimum and maximum values and the crosses represent the outliers.

There was no significant difference in the distribution of all groups of cells between implanted and contralateral regions among time points evaluated (Kruskal-Wallis - Bonferroni *post hoc* test; p>0.05) (Fig 5).

### Neuronal morphology

**Fig 6:**
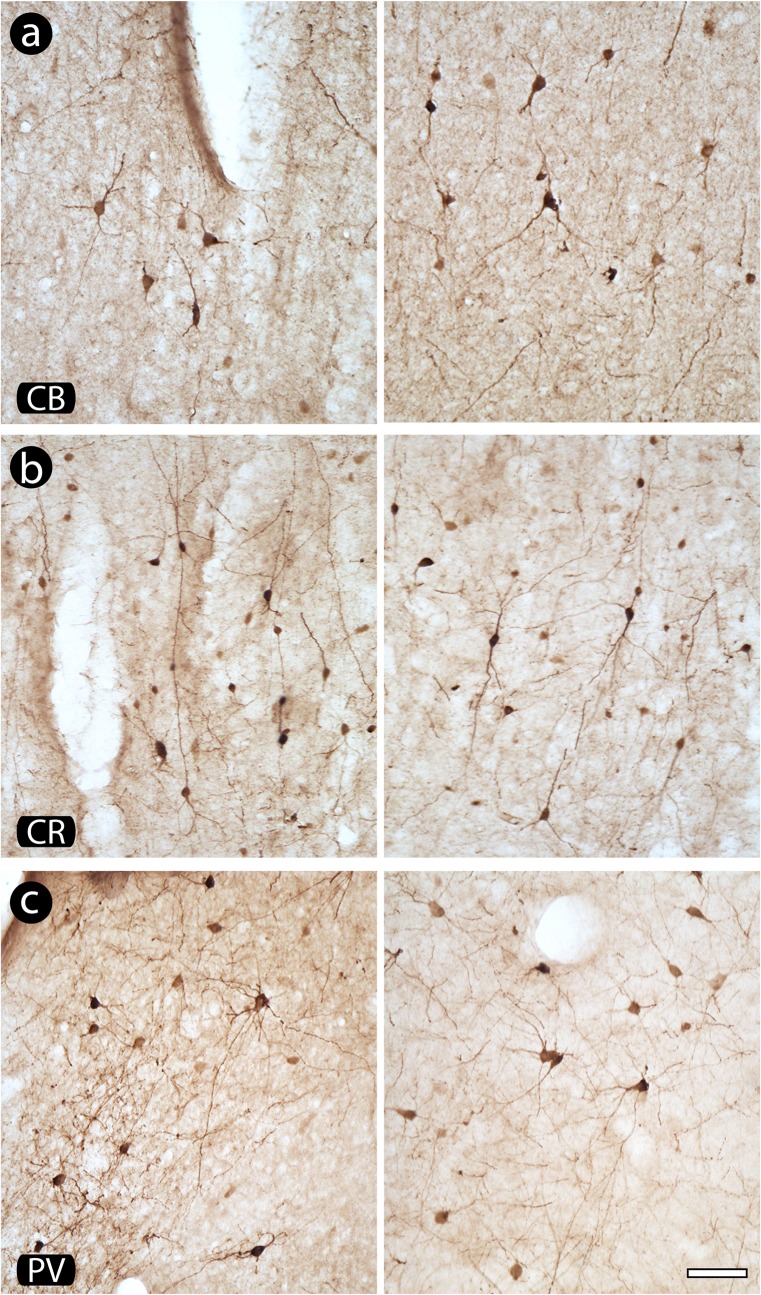
Morphology of neurons reactive to calcium binding proteins (CBP) in implanted and contralateral hemispheres. Notice that there is no evidence of alteration in both morphology and pattern of reactivity of calbindin (CB)- (a), calretinin (CR)- (b) and parvalbumin (PV)-positive neurons (c) when implanted and contralateral hemispheres are compared. Left column of the figure: implanted hemisphere; right column of the figure: contralateral hemisphere, adopted as control. Scale bar: 50 μm.

Chronic implants did not induce morphological alterations on inhibitory cells across implanted cortex. A qualitative analysis revealed a normal reactivity of CBP-reactive neurons (see examples in Fig 6).

In general, CB-highly-reactive neurons from supragranular layers had fusiform or round cell bodies, with bipolar or multipolar dendritic arborizations (Fig 6A). Neurons found in the infragranular layers were typically multipolar, with oval/round cell bodies. Less-reactive neurons from supragranular layers were normally multipolar, presenting fusiform or oval cell bodies and a proximal dendritic arborization.

The CR-reactive neurons located in supragranular layers possessed bipolar or bitufted vertically oriented somatodendritic morphology (Fig 6B), with unequivocally labeled dendrites originating from the opposite cellular poles, one of them projecting towards layer I and other projecting to layers V/VI, possessing a fusiform or oval cell body. Besides these bipolar neurons, other morphological types of neurons, mainly multipolar cells, were observed. Horizontal cells were rarely found.

PV-immunoreactive cells resembled a multipolar dendritic arborization, with an oval cell body, especially in the infragranular layers, although a few bipolar cells could also be discerned. In the supragranular layers the majority of the cell bodies were fusiform, with a multipolar dendritic arborization emerging from the cell body (Fig 6C).

### Microglial response

**Fig 7:**
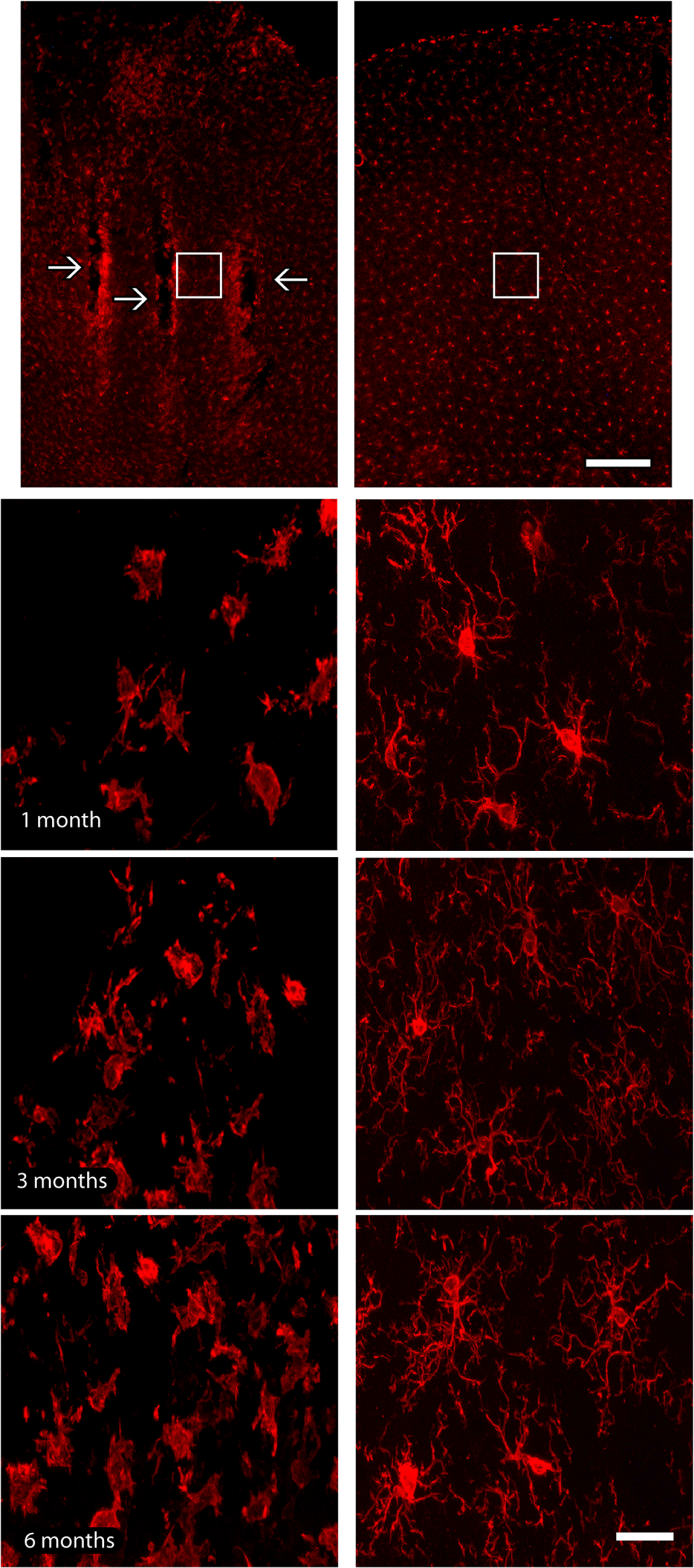
Microglial immunoreactivity across electrode tracks. (a). Electrode implantation sites (arrows) were identified as a well-defined strip of microglial labeling, especially in latter survival time point. (b). Iba1-reactive microglial cells with a hypertrophic morphology were observed only in the vicinity of implants, whilst microglial cells presented a non-activated morphology in the contralateral hemisphere, possessing stellate non-hypertrophic cell bodies and thin and elongated processes. Activated microglial cells were not observed far from implant sites neither in contralateral hemispheres. White squares in lower magnification pictures indicate the regions where enlargements were obtained in all groups. Left column of the figure: implanted hemisphere; right column of the figure: contralateral hemisphere, adopted as control. Scale bar: 100 μm (lower magnification); 25 μm (enlargements).

Microglial activation was observed around the electrode tracks, mainly after 6 months of implant, appearing as a stripe of labeling circumscribing the implant site (Fig 7A). Activated microglial cells around implanted regions were characterized by hypertrophic cell bodies and thicker and shortened processes, whilst in the contralateral hemisphere microglial cells possessed a non-activated morphology, with cells presenting stellate non-hypertrophic cell bodies and thin and elongated processes (Fig 7B). Activated microglial cells were not observed far from implant sites neither in contralateral hemispheres, suggesting that electrode implants did not alter the physiology of adjacent cortical regions.

## Discussion

In the present work we characterized the impact of chronic implanted tungsten multielectrode arrays on the distribution and morphology of some classes of inhibitory neurons across the rat’s motor cortex. In addition, to estimate the impact of post-implant elapsed time on neuronal recordings, we also evaluated the temporal variation of electrophysiological signals. We obtained three main findings. First, our results reinforce the notion that chronic microelectrode arrays are well tolerated by the rat cortex, with electrodes remaining functional until 6 months after implantation. Recordings exhibited a peak of high signal quality around 3 months of implant. Second, both morphology and distribution of inhibitory neurons were not affected by these chronic implants. Third, both a restricted tissue loss and microglial activation were observed solely immediately around the microwire penetration tracks.

### Tissue integrity and variation of neuronal signal after chronic implants

Currently one of the main goals of the field of neuroengineering is the development of stable and compatible devices to be incorporated to the human body. The capacity of an implanted prosthesis to coexist harmonically with the host tissue without causing destructive changes is defined as biocompatibility. In line with this, distinct types of multielectrode arrays have been tested in order to evaluate their efficiency and tolerability by the nervous system [27–33].

Over the past two decades, the efficiency of stainless steel and tungsten microwires, clustered in bundles or arrays, to simultaneously record neuronal populations throughout several months and even years has been demonstrated [34–37]. Yet, it is still essential to perform a general characterization of the impact of the long-term presence of these microelectrodes in brain parenchyma, and also evaluate how the relationship neuronal signal/tissue preservation influences motor and cognitive tasks. In a previous study we described a small degree of tissue alteration induced by the presence of chronically implanted tungsten microelectrode arrays, including a small loss of tissue reactivity restricted to the sites of electrode implantation, and a minimal cell death around electrode tracks. Such alterations, however, did not implicate in any measurable function loss since normal physiological neuronal responses were obtained at the implanted sites [22]. Here we used a microwire somewhat thicker than our previous report (50-μm *versus* 35-μm) [22], with analogous results regarding tissue response, including a small loss of tissue and a restricted microglial reaction across implantation sites, what reinforces the notion that such type of array design is well tolerated by the tissue even with relatively thicker wires.

Gliosis is a physiological event triggered by minimal alterations on the cellular environment, in order to safeguard normal tissue against additional wound [38], being defined as a pathological hallmark of CNS structural injuries [39]. After a damaging event, astrocytes and microglial cells respond quickly, retracting their extensions and assuming an amoeboid-like form, originating the so-called glial scar [40]. Formation of the glial scar around the microelectrodes insulates them from nearby neurons. This is an important event in the early stages of the reaction to the microelectrode implantation because it leads to an initial improvement of extracellular signal’s selectivity [41]. Conversely, encapsulation increases the microelectrode’s impedance over time. Such event may underlie two antagonic processes, which take place at different moments in time. Initially, it may help increase the SNR, contributing to a higher neuronal yield during the first few months of the implant. Later on, however, further encapsulation of the microelectrode tip may contribute to a reduction in neuronal signal clarity [42]. This latter event might explain the decrease of neuronal signal strength we observed 6 months after implantation, given that the cortical tissue remained physiologically functional even at later stages of chronic microwire presence. In congruence with this, in a recent study our group showed that astrocytic activation increased after 6 months of implantation of 50μm electrode array as compared to 1 and 3 months of implant, but only in the region circumscribing the implantation site, defined by a cell body hypertrophy and thickening and shortening of processes [11]. In the same token, Prasad et al. (2012) described a large variation in impedance, performance, and structural changes at the microelectrode recording surface in the first 2–3 weeks post-implant, associated to a further decay of neural signal over time [43]. As both gliosis and inflammation will always occur after any tissue disturbance [44–46], including chronic electrode implantation, selective control of this response becomes important, mainly because uncontrolled or generalized microglial/astroglial activation induces the generation of pro-inflammatory cytokines, such as tumor necrosis factor alpha (TNF-α), interleukin-1 beta (IL-1β), and nitric oxide (NO). These substances are all potentially harmful to nervous tissue [47,48], since they can contribute to the cell death around electrodes and, hence, total failure of recordings. However, an infiltration of peripheral macrophages after tissue disturbance cannot be entirety ruled out. Additional studies seeking for a refined identification of distinct types of immunoreactive glial cells are needed to clarify this issue.

### Electrode implants and interneurons

Although GABAergic interneurons constitute a minority population within the brain, these cells play a pivotal role, seeing that they are crucial for the control of inhibition (see [49–51] for reviews). Its resistance to injury is variable, depending especially on the degree and location of the lesion. Some studies point to a relative preservation of this cell group after ischemic injury and heavy metal intoxication [52,53] whereas others indicate a severe degeneration of interneurons following excitotoxicity and acute seizure [54,55]. Interneuron failure is directly involved with important brain disturbances such as schizophrenia and bipolar disorder [56,57]**,** since a minimal disinhibition of the excitatory neurons induced by a breakdown of the inhibitory input from the GABAergic cells results in a hyperexcitability status [58].

Inhibitory interneurons display a large diversity in both their biochemical patterns and spike firing properties, what concurs to define their discrete subpopulations [59,60]. For instance, distinct classes of interneurons can be identified according to what CBP they contain. CBP major role is to buffer and transport free calcium [61]. The control of calcium levels in the nervous tissue is particularly important because cellular degeneration is widely associated to its overload, which triggers the activation of intracellular processes leading to failure of organelles and proteins and lipids, ultimately resulting in cell death [48]. Since neurons containing CBP have a greater capacity to buffer calcium, these cells might be more resistant to degeneration induced by the excessive presence of this ion. In support to this notion, previous studies have shown that CBP-reactive cells are less vulnerable to degeneration in altered states such as excitotoxicity, epileptic status and Parkinson’s disease [62–65], although some works challenge this concept [66,67]. In a study evaluating CBP expression following traumatic brain injury in humans, Buritica et al. (2009) reported a variation in both reactivity and number of CPB-reactive neurons depending on the region evaluated (epicenter of the lesion or region of traumatic penumbra) **[68]**. Neurons found in penumbral region were relatively better preserved than those from the region primarily affected, indicating that expression of CBP is related to temporal modifications following injury, affecting the regulation of cortical excitability throughout the evolution of a severe lesion.

In the present work we observed that both the morphology and general distribution of CBP neurons were not significantly affected by the chronic implant of tungsten microwire arrays. Such result can be explained in part by the small mechanic lesion caused by the electrodes, which results in a minimal alteration in the nearby cortical cellular milieu. Given that, as previously stated, CBP cells act as calcium buffers, incidental modifications in the levels of this ion around implanted tissue could be kept under control by these interneurons [69]. In addition, microglial activation observed around implanted sites likely helps to maintain the physiology of the cellular environment after excitotoxic events (increase of free glutamate and calcium in the cortical parenchyma following the implantation of microwires) [70]. Taken together, these factors may contribute to the preservation of the integrity of the CBP interneurons even 6 months after multielectrodes array implantation.

### Conclusions

During the last years a considerable progress has been achieved on BMI technology. The refinement of the techniques employed to the development of cortical neuroprosthetic devices is an extremely relevant point for the improvement of the quality of life of people suffering from restrictive body paralysis. A proper understanding of how brain responds to invasive techniques is critical, especially concerning to the preservation of the tissue functionality. As an extension of previous studies of our group [11,22], here we aimed to contribute with some additional data to this topic, by evidencing that CBP-reactive neurons were not impacted by chronic implant of tungsten arrays. Yet, more studies involving the analysis of neuronal morphology of excitatory and inhibitory cells in other cortical and subcortical areas are required in order to provide additional information to be employed on the achievement of an effective BMI rehabilitation apparatus.
